# The Multifaceted Role of GPCRs in Amyotrophic Lateral Sclerosis: A New Therapeutic Perspective?

**DOI:** 10.3390/ijms23094504

**Published:** 2022-04-19

**Authors:** Davide Bassani, Matteo Pavan, Stephanie Federico, Giampiero Spalluto, Mattia Sturlese, Stefano Moro

**Affiliations:** 1 Molecular Modeling Section (MMS), Department of Pharmaceutical and Pharmacological Sciences, University of Padova, 35131 Padova, Italy; davide.bassani.1@studenti.unipd.it (D.B.); matteo.pavan.7@phd.unipd.it (M.P.); mattia.sturlese@unipd.it (M.S.); 2 Department of Chemical and Pharmaceutical Sciences, University of Trieste, 34127 Trieste, Italy; sfederico@units.it (S.F.); spalluto@units.it (G.S.)

**Keywords:** amyotrophic, sclerosis, ALS, GPCR, adenosine, serotonin, histamine, glutamate, cannabinoid, adrenergic

## Abstract

Amyotrophic lateral sclerosis (ALS) is a degenerating disease involving the motor neurons, which causes a progressive loss of movement ability, usually leading to death within 2 to 5 years from the diagnosis. Much effort has been put into research for an effective therapy for its eradication, but still, no cure is available. The only two drugs approved for this pathology, Riluzole and Edaravone, are onlyable to slow down the inevitable disease progression. As assessed in the literature, drug targets such as protein kinases have already been extensively examined as potential drug targets for ALS, with some molecules already in clinical trials. Here, we focus on the involvement of another very important and studied class of biological entities, G protein-coupled receptors (GPCRs), in the onset and progression of ALS. This workaimsto give an overview of what has been already discovered on the topic, providing useful information and insights that can be used by scientists all around the world who are putting efforts into the fight against this very important neurodegenerating disease.

## 1. Introduction

Amyotrophic Lateral Sclerosis (ALS, also referred to as “motor neuron disease”) indicates a clinical situation in which the motor neurons of patients undergoa progressive loss in their function and number [1]. This type of neuronal cell, whose cell body is localized in the motor cortex, the brainstem, and the spinal cord, is responsible for the innervation and the control of muscle fibers, essential for voluntary muscle contraction [2]. Their loss has very important consequences on the patient’s life, firstly impairing the ability to chew and walk, then to speak and to move, until even the ability to breath is affected, leading, after 2–5 years, to death due to respiratory failure [3]. ALS can be classified into two main types, “sporadic ALS” (the great majority of all cases), which has no known cause and typically has its onset between the ages of 58 and 63 years, and “familial ALS” (about 5–10% of cases), which is linked to genetic factors, and has its onset between the ages of 47 and 52 years [4].

In both scenarios, the pathology starts with the manifestation of muscle weakness and atrophy, with methods and timing very variable based on the patient and on the parts of the motor neurons that are affected first [5]. Indeed, a classification of the onset of the pathology can be made with reference tothe site of its onset. For two-thirds of patients, the limb muscles are affected first (“spinal ALS”), with manifestations mainly in the distal muscles of the dominant hand for the upper limb and in the hamstrings for the lower limb. For the greater part of the remaining patients, the bulbar muscles represent the onset site (“bulbar ALS”), and in this case, dysphagia and chewing problems represent the first manifestations of the pathology [6].

The next steps of the diseaseinvolve the progressive spreading of the neurodegenerationprocess to the unaffected motor neurons, causing an increasing worsening in the patient’s daily life, making activities such as eating and walking continuouslymore difficultand leading to their complete loss. The final and worst clinical scenario has its onset when the respiratory function is significantlyaffected, progressively increasing the risk of respiratory failure, which is the main cause of death due to ALS [7].

**Figure 1:**
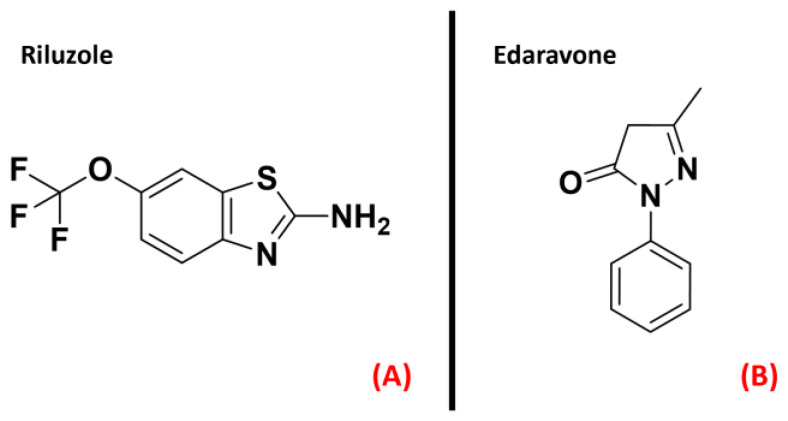
The chemical structures of Riluzole (**A**) and Edaravone (**B**),which are the only two small-molecule drugs approved currentlyfor ALS treatment.

Even if much effort has been madeamongboth academic and industrial scientific groups, no cure has yetemerged for ALS. Riluzole [8] and the recently FDA-approved drug Edaravone [9] (both represented in Figure 1) constitute the only two small molecules used for the ALS treatment, and only succeed in slowing down the disease’s progression [10].

Such a neurodegenerating process, affecting 1.75–3 people per 100,000 [11], has, in the great majority of cases, no known cause [12], making it even harder todesign a therapy for this disease. From a biochemical point of view, the hallmark of ALS is considered to be the presence of inclusion bodies in the cytoplasm of motor neurons. These aggregates are formed by the TAR DNA-binding protein 43 (TDP-43) [13], a protein involved in several important physiological functions such as DNA repair, splicing, and transcriptional regulation. Even if its main localization site is the nucleus, processes such as its hyperphosphorylation or the mutation of its gene (TARDBP) lead to its aggregation in the cytoplasm [14]. This mislocalization directly causes the dysregulation of several cellular events related toRNA metabolism, DNA replication, and oxidative stress management, leading to the loss of the motor neurons affected [15]. Other molecular targets thathave been demonstrated to be important for ALS onset and progression are superoxide dismutase (SOD1) [16] and DNA/RNA-binding protein FUS/TLS (FUused in Sarcoma/Translocated in LipoSarcoma, also called “FUS”) [17], which appear to be mutated in the patients.

The knowledge that hyperphosphorylation of TDP-43 is one of the main processes leading to its aggregation has led the scientific community to devotesome efforttoidentifying the protein kinases responsible for such processes, in order to find proper inhibitors for such species [18]. Recent work by Guo et al. goes deep in the examination of the involvement of kinases in ALS progression, enucleating species such as CK1, ERK, GSK3β, and JAK3 as promising targets for the treatment of this neurodegenerating disease [19]. Another article by Palomo et al. gives an exhaustivepanoramic view of the protein kinase inhibitors currently in clinical trials for ALS treatment [20]. Riluzole has proven to increase life expectancy by about 2–3 months [21], and even if its main target still remains the NMDA receptor, recent work by Bissaro et al. suggestedthat this mechanism could be due to its action on the delta isoform of CK1 [22].

Many molecular candidates (both new chemical entities and compounds coming from repurposing strategies) are nowadays in clinical trials for ALS [23], acting on different biological pathways, with the common aim being to restore the neuronal health status in the affected patients, possibly trying to go in the direction to find a proper cure for this pathology [24]. A comprehensive list of the potential small-molecule drugs now being evaluated by the FDA in clinical phases is reported in Table 1.

Even if some effort has been directed toward trying to highlight the role of protein kinases in ALS progression, this has not been recently orextensively done with respect toG-protein coupled receptors, biological actors which have been demonstrated to be detrimental to neuronal and physiological conditions.It is important to remember that ALS is a non-cell-autonomous disease, which means that the neuronal damage characterizing the pathology is caused by aberrant processes also happening outside the neurons themselves. Indeed, ALS progression has been demonstrated to be strongly related to glial cell dysregulation (mainly microglia and astrocytes) [25]. GPCRs are very widely expressed proteins in the human organism [26], and so a beneficial effect could also be obtained by targeting extraneuronal receptors, which could trigger biological processes that, in the overall scenario, could mitigate if not reverse the disease progression.

GPCRs are membrane receptors and constitute one of the main protein families encoded by human genes, with more than 800 members already identified [27], divided intosixdifferent classes (identified alphabetically with letters from “A” to “F”) based on their similarities in sequence and function. They all share a common architecture formed of a seven-α-helixtransmembrane domain (usually referred to as “7-TM”), an extracellular N-terminal domain, and an intracellular C-terminal domain. These proteins exert their roles by coupling with an intracellular messenger called “heterotrimeric G protein”, which is formed by α, β, and γ subunits, and interacts with different intracellular partners based on its type. The α subunit is displaced from the βγ-complex upon GPCR–ligand binding, and its fate depends on its Gαfamily belonging. Indeed, activated G_i/0_ proteins inhibit adenylyl cyclase (AC), reducing the production of the second messenger cyclic adenosine monophosphate (cAMP); G_s_, conversely, activates adenylyl cyclase, and G_q_αsubunits activate phospholipase C (PLC), leading to an increase in Ca^2+^ influx in the cytoplasm [28].The physiological roles of GPCRsinclude homeostasis modulation, mood balancing, immune system regulation, neuronal plasticity, and many more [29]. The goal of the present work is to give a panoramic view of the GPCRs which have been linked to ALS onset and progression, presenting what has already been done to modulate their action, and highlighting new potential therapeutical scenarios. Table 2 summarizes the outcomes of our study, listing the GPCR targets that will be discussed and highlighting the new possible paths that can be taken in order to exploit their therapeutic potential for ALS.

Our work will be beneficial for all of the scientists whoare dedicating their knowledge and efforts to the eradication of ALS.

## 2. GPCRs Involved in ALS

### 2.1. Puringergic Receptors P2Y and Adenosine Receptor A_2A_AR

Purinergic receptors are a peculiar class of membrane receptors, sensitive to a wide series of purinergic ligands such as ATP, ADP, UTP, UDP, UDP-glucose, and adenosine. The kind of molecules interacting with them defines their classification intoone of the three subfamilies forming this class. The first group, called “P1 receptors”, is formed of GPCRs activated upon adenosine binding (and for these reasons are also known as “adenosine receptors”), while “P2Y receptors” are GPCRs that can bind to ATP, UDP, and their diphosphate analogs ADP and UDP (with the addition of UDP-glucose). The last subfamily, named “P2X receptors”, are ligand-gated ion channels exclusively sensitive to ATP [95]. Being the P2X family not formed by GPCRs, our evaluations will focus on the first two families of receptors.

**Figure 2:**
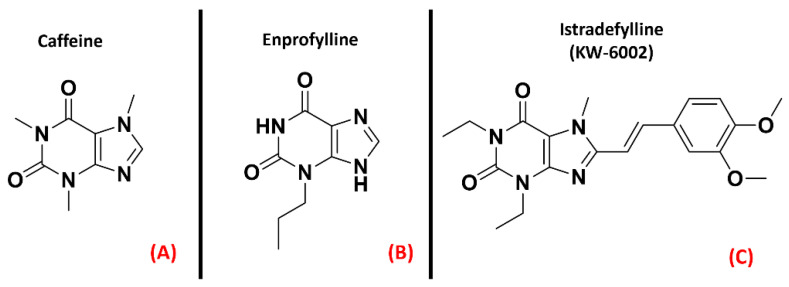
The chemical structures of the non-selective adenosine receptors antagonistscaffeine (**A**) and Enprofylline (**B**), and the selective A_2A_AR antagonist Istradefylline (**C**).

The “P1” subfamily, more commonly referred to as adenosine receptors (ARs), is a group of purinergic class AGPCRs divided into foursubtypes, A_1_AR, A_2A_AR, A_2B_AR, and A_3_AR, each involved in many different physiological processes. While the functions of A_2B_AR and A_3A_R are mainly related to the circulatory, immune and respiratory systems, the A_1_AR and A_2A_AR proteins are importantly present in the central nervous system (CNS) [96]. Moreover, the A_1_AR and the A_2A_AR receptors have been demonstrated to play a crucial role in neuroprotection, neuronal survival, and neuroinflammation [30]. A study from Vincenzi et al. reported an upregulation of A_2A_AR receptors in the lymphocytes of people affected byALS [31], while Yoshida et al. measured adenosine levels in the cerebrospinal fluid of ALS patients, finding out that these were significantly higher withrespect to the control subjects [32]. Unexpectedly, treatment with the A_2A_AR antagonist caffeine (Figure 2, panel A), which is usually referred to as a protective agent against Alzheimer’s Disease (AD) and Parkinson’s Disease (PD), was demonstrated to shorten the survival of ALS-affected SOD1^G93A^ mice, a well-known experimental model for ALS (Potenza et al.) [33], even if some explanation for this phenomenon can be provided bythe non-selectivity of caffeine [34]. Indeed, Ng et al. showed that suppression of A_2A_AR signaling delays the progression of ALS in the same SOD1^G93A^ mouse model [35]. This is in accordancewith the study of Mojsilovic-Petrovic et al., which demonstrated that A_2A_AR inhibitors (such as the non-selective enprofylline(Figure 2, panel B), and the A_2A_AR-selective KW-6002(Figure 2, panel C), also called Istradefylline) protectmotor neurons from toxic insult, highlighting the beneficial effects of such activity for ALS patients [36].

**Figure 3:**
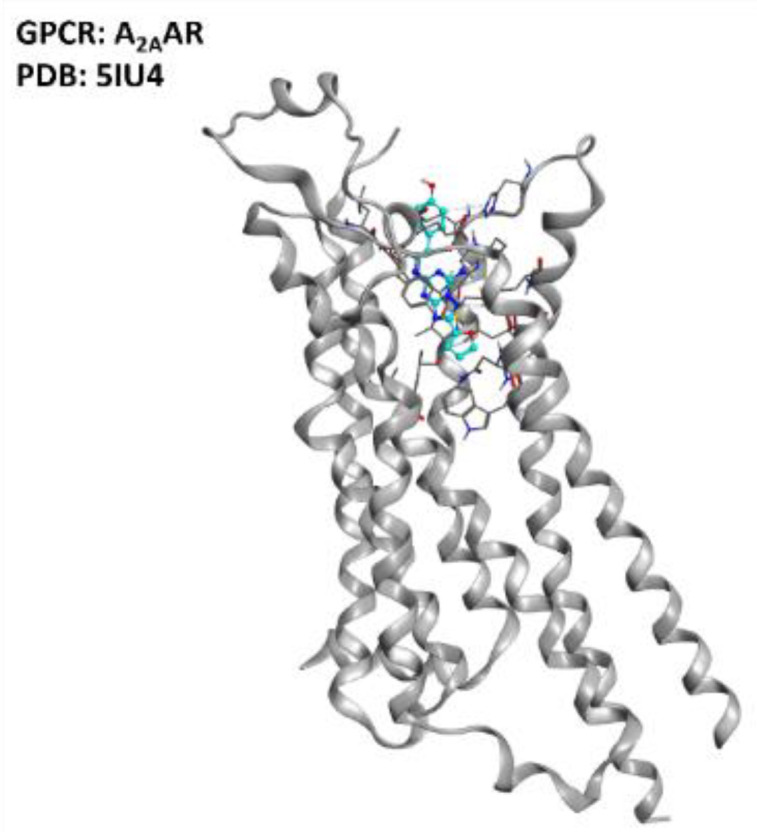
Representation of the structure of the A_2A_AR receptor (sourced from the Protein Data Bank [97], PDB code: 5IU4 [98], method: X-ray diffraction, resolution: 1.72 Å). The image was created and rendered with the Molecular Operating Environment (MOE) suite [99].

Despite all this evidence, a study from Liu et al. showed that A_2A_AR activation, suppressing AMPK activation, suppressed TDP-43 mislocalization [37]. The multifactorial nature of ALS makes it very difficult to define sharply whether agonism or antagonism of the A_2A_AR receptor has the best risk/benefit ratio, but the literature clearly defines this GPCR (represented in Figure 3) as one of the promising targets for ALS treatment.

Thesecond subfamily of purinergic receptors, “P2Y”,ispresent in a great variety of human tissues, but their main biological roles are identifiable in blood clotting, vasodilatation, and immune response [100]. The P2Y family comprises eightdifferent isoforms, among which P2Y_12_ has gained the interest of the scientific community for its role in neuroinflammation, as addressed by Morillas et al. [38] and Amadio et al. [39].

**Figure 4:**
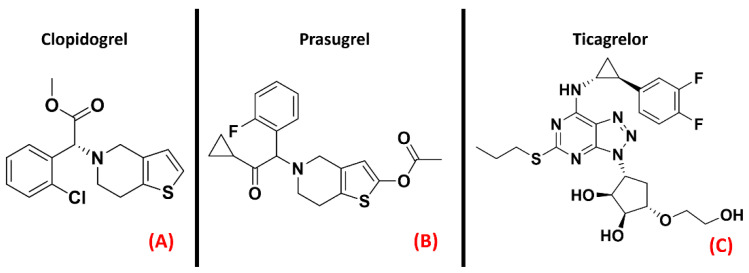
The chemical structures of the selective and irreversible P2Y_12_ receptor antagonists Clopidogrel (**A**) and Prasugrel (**B**). Ticagrelor, a selective, reversible, allosteric P2Y_12_ receptor antagonist, is also reported (**C**).

Jacobson et al. recently highlighted the proinflammatory effect of P2Y agonists, reporting that antagonizing this class of GPCRs could be considered a way of treating inflammatory conditions [40]. Even if inhibitors of the P2Y_12_ isoform are already marketed as antiplatelet drugs (e.g., Clopidogrel, Prasugrel, Ticagrelor, all represented in Figure 4), Jacobson et al. pointed out that there is a lack of selective and versatile P2Y ligands for each subtype, meaning thatthe drug discovery process is still very active in this specific field.

**Figure 5:**
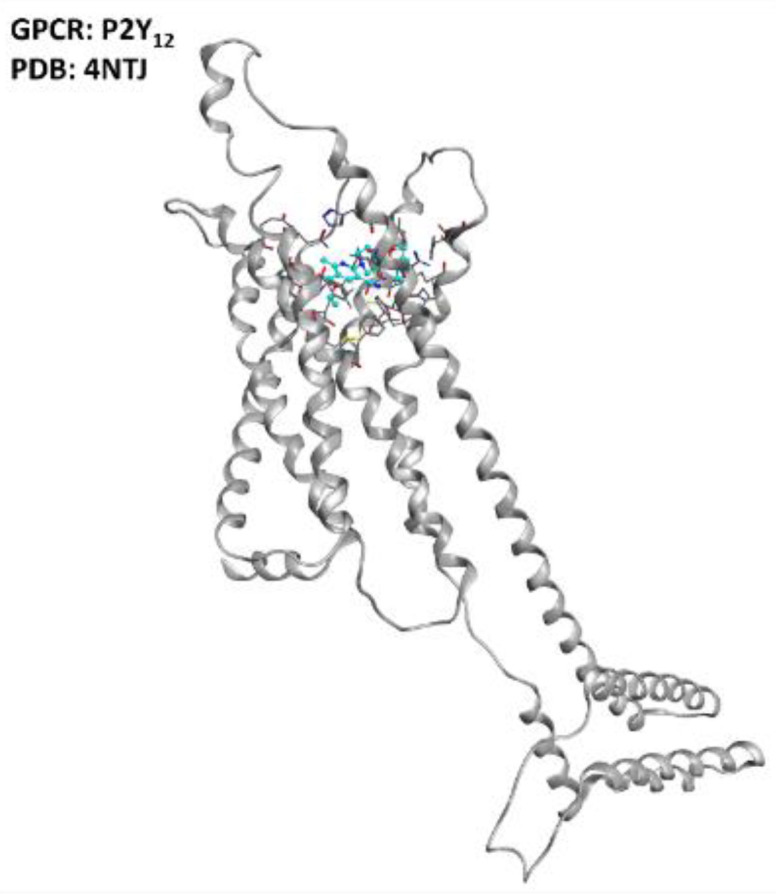
Representation of the structure of the P2Y_12_ receptor (sourced from the Protein Data Bank, PDB code: 4NTJ [101], method: X-ray diffraction, resolution: 2.62 Å). The image was created and rendered with MOE.

Specifically, D’Ambrosi et al. reported an upregulation of P2Y_6_ receptors in the microglia SOD1 mutant models of ALS, also remembering that this phenomenon is associated with brain damage [41]. Moreover, P2Y_12_ (represented in Figure 5) is upregulated in spinal cord microglia upon nerve injury, as pointed out by Kobayashi et al. [42]. Converging information is provided by a study from Moore et al. [43], further confirming P2Y_12_ as a potential target for modulating neuroinflammation and neuronal damage. The data currentlyavailable help in suggesting the practical possibility of ALS regulation through purinergic receptor modulation, and this will be realizable as soon as proper inhibitors canbe designed, potentially avoiding the non-desired antiplatelet effect of these molecules.

### 2.2. Chemokine Receptors

**Figure 6:**
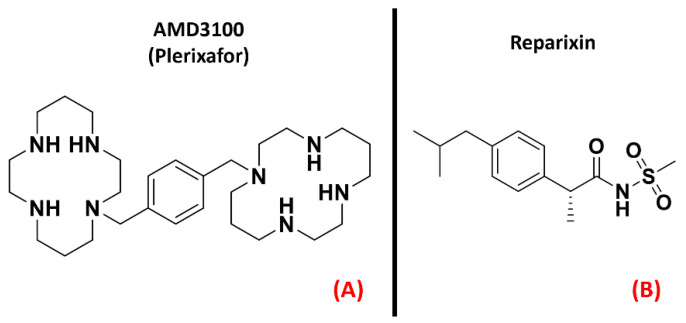
The chemical structures of the CXCR4 antagonist AMD3100 (also known as “Plerixafor”, (**A**) and the inhibitor of CXCR1 and CXCR2 known as Reparixin (**B**).

Chemokine receptors constitute a group of about 20 classes of GPCRs found mainly on the surface of leukocytes, which respond to specific ligands to control chemotaxis. The ligands for these proteins are called chemokines and are a peculiar kind of cytokine used for inducing a directional movement of certain types of cells, such as epithelial and immune ones. The chemokines, as well as the receptors they act on, can be divided into fourfamilies, namely CC (e.g., chemokine CCL4), CXC (e.g., chemokine CXCL8, also known as IL-8), XC (e.g., XCL1), and CX3C (of which the only member today is CX3CL1, also called neurotactin) [102]. The activation of chemokine receptors leads to Ca^2+^ influx and cell mobilization [103]. Several of these receptors are important in the progression of motor neuron damage. La Cognata et al. highlighted an upregulation of CXCR2 in both sporadic ALS patients and SOD1^G93A^ mice, showing that treating the mouse models with the CXCR2 allosteric inhibitor Reparixin (Figure 6, panel B), the neuromuscular function of the subjects was improved [44]. Another interesting paper published by Rabinovich-Nikitin et al. highlighted the benefits in terms of lifespan and motor function obtained on SOD1^G93A^ mouse models through the administration of the CXCR4 antagonist AMD3100 (also known as “Plerixafor”, Figure 6, panel A) [45].

**Figure 7:**
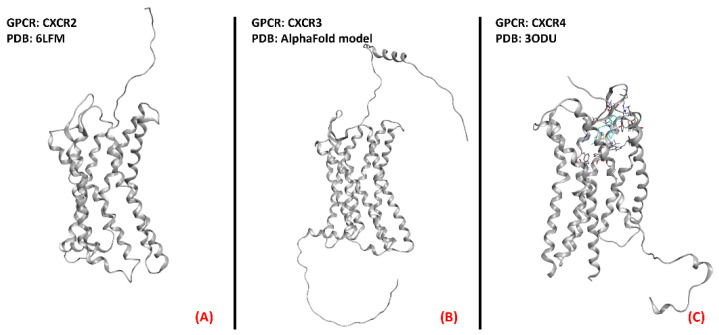
Representation of the three-dimensional structures of the chemokine receptors that could be considered for ALS treatment. CXCR2 (**A**) receptor (sourced from the Protein Data Bank, PDB code: 6LFM [104], method: cryo-EM, resolution: 2.50 Å), CXCR3 (**B**) (with no experimentally resolved structure available, the model from the AlphaFold [105] database is presented), and CXCR4 (**C**) receptor (sourced from the Protein Data Bank, PDB code: 3ODU [106], method: X-ray diffraction, resolution: 2.50 Å).The images were created and rendered with MOE.

Several scientific works have reportedan increase in circulating chemokines and cytokines in ALS patients, as recently detailed by Liu et al. [46], and the upregulation of chemokine receptors CXCR3, CXCR4, and CCR2 wasalso highlighted in the pathology of interest by Perner et al. [47], whoalso proposed CXCR3 and its ligands as possible therapeutic targets for ALS. These scientific works converge in addressing chemokine receptor modulation as a possibility for ALS treatment, focusing on the antagonism of certain isoforms.The three-dimensional structures of CXCR2, CXCR3, and CXCR4 are represented in Figure 7.

### 2.3. Angiotensin II Receptors (ATRs)

**Figure 8:**
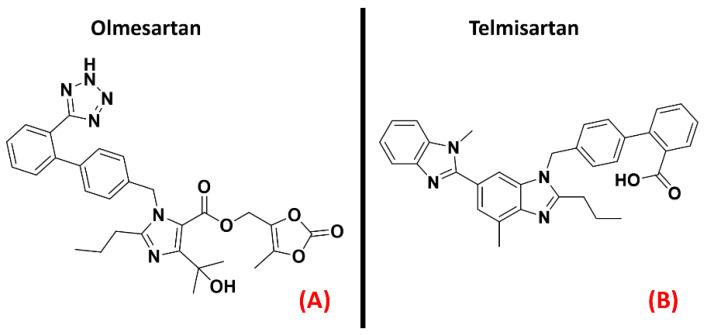
The chemical structures of the AT_1_ antagonists Olmesartan (**A**) and Telmisartan (**B**).

Angiotensin II receptors (ATRs) are a group of GPCRs that have gained fame for their importance in the therapy of hypertension. Indeed, their main physiological role is related to the renin–angiotensin–aldosterone system, one of the main physiological pathways for blood pressure regulation and fluid and electrolyte balance [107]. Briefly, the peptide hormone angiotensinogen is secreted by the liver and cleaved by renin to form angiotensin I, which is then converted to angiotensin II by the angiotensin-converting enzyme (ACE), produced by the lungs. Angiotensin II acts on its receptors and modulates several processes, such as aldosterone secretion (in the adrenal glands), water and sodium retention (in the kidneys), sanguine pressure, and vasopressin production (in the CNS) [108]. To treat hypertension, many efforts have been directed towards the creation of drugs acting as ATRs inhibitors. The most famous drug family designed for this purpose is represented by the “Sartans”, which selectively bind to the first isoform of angiotensin receptors [109]. Indeed, ATRs can bedivided into fourisoforms, AT_1_, AT_2_, AT_3_, and AT_4_. While the lattertwo are still in the early stagesof research, the first two isoforms have been more deeply characterized. AT_1_ is mainly found in blood vessels, heart, kidney, brain, and adrenal cortex, mediating vasoconstrictive effects [107]. AT_2_ receptors are more concentrated in the fetus and neonate, and their functions are more strongly related to neuronal development and excitability [110]. A study byKawajiri et al. highlighted a reduction in angiotensin II levels in the CSF coming from ALS patients, reporting two opposite consequences:the reduction of protection and repair mediated by AT_2_, on the one hand, and the reduction of oxidative stress due to AT_1_ on the other. Indeed, Kawajiri et al. hypothesized that angiotensin II could be downregulated in CSF of ALS patients as a protective reaction, avoiding excessive activation of AT_1_ [48]. The benefits of AT_1_ antagonism have also been underlined by Iwasaki et al., whoreported evidence of the neurotrophic effects on spinal motor neurons of the drug Olmesartan (Figure 8, panel A), specifically referring to its potential application in ALS [49]. Furthermore, an article by Mammana et al. highlighted the AT_1_ antagonism-mediated neuroprotective effects of Telmisartan (Figure 8, panel B), also outlining the decrease in neuronal injury and microglial activation causedby it [50].

**Figure 9:**
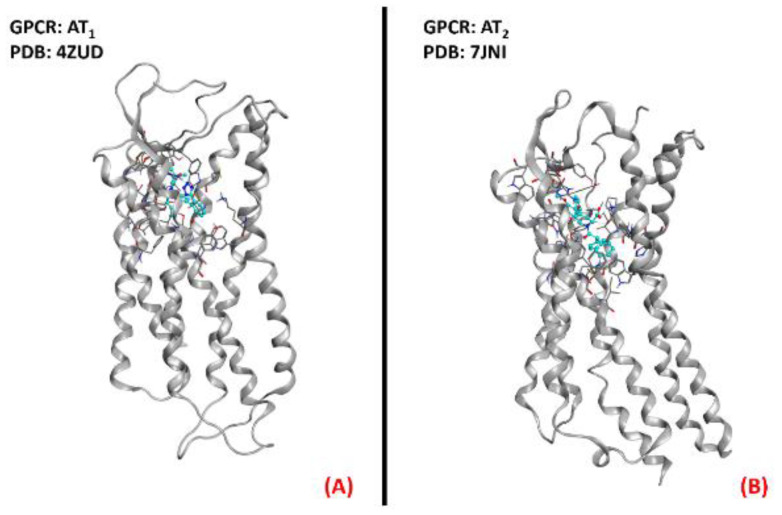
Representation of the three-dimensional structures of the angiotensin II receptors thatcould be considered for ALS treatment. AT_1_ receptor (**A**) (sourced from the Protein Data Bank, PDB code: 4ZUD [111], method: X-ray diffraction, resolution: 2.80Å) and AT_2_ receptor (**B**) (sourced from the Protein Data Bank, PDB code: 7JNI [112], method: X-ray diffraction, resolution: 3.00 Å). The images were created and rendered with MOE.

Summing up the information obtainable from the literature, AT_1_ inhibition could be examined as a potential new therapeutic method of fighting ALS conditions. Both AT_1_ and AT_2_ receptors are represented in Figure 9 below.

### 2.4. Dopamine Receptors

Dopamine receptors are among the most important and widely studied G-protein coupled receptors, mainly for their important physiological roles in neurotransmission. This class of GPCRs is divided into five isoforms, which are separated into two classes. The first, also called the “D1-like family”, comprises the D1R and D5R receptors, which are coupled to a G_s_ protein responsible for adenylyl cyclase activation upon binding. The second family, also known as the “D2-like” family, comprises the D2R, D3R, and D4R proteins, all coupled with a G_i_ protein with inhibitory activity on adenylyl cyclase. Dopamine receptors are localized in different peripheral parts of the organism, such as arteries, heart, and kidneys, but their activities much more determinant within the CNS. Indeed, dopamine is the main neurotransmitter involved in the reward system, and its signaling is of crucial importance for processes such as cognition, memory, and motor control [113]. Dysregulation of the dopaminergic system in the brain represents the main cause of very important diseases such as schizophrenia, attentiondeficit hyperactivity disorder (ADHD), and Parkinson’s disease [114]. Several articles highlight a correlation between dopamine signaling and ALS development [51]. We have previously reported what was assessed by Liu et al. regardingthe protective effect of the A_2A_ receptor on TDP-43 mislocalization [37]. A recent study by Lai et al. details how this beneficial activity can be blocked by D2R activation [52]. Despite this, D2R was also identified as important for the modulation of motor neuron excitability by Huang et al. [53]

**Figure 10:**
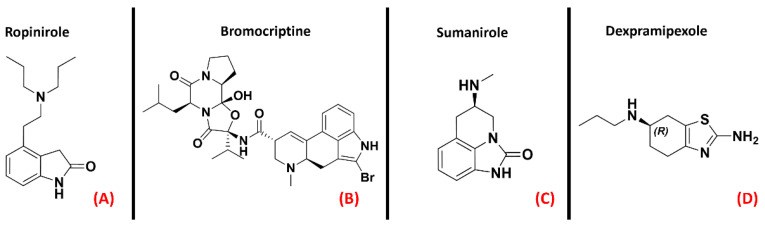
The chemical structures of the dopamine receptor agonists Ropinirole (**A**), which has an affinity for D2R, D3R, and D4R, Bromocriptine (**B**), also non-selective with an affinity for D2R, D3R, and D4R, and Sumanirole (**C**), selective for D2R. (**D**) Chemical structure of Dexpramipexole (its neuroprotective effects are attributed to dopaminergic-independent activities).

Fujimori et al. showed how the treatment with Ropinirole (Figure 10, panel A), an agonist for receptors D2R, D3R, and D4R mainly used for Parkinson’s disease, has neuroprotective effects in ALS models [54]. Additionally, D2R agonists such as Bromocriptine and Sumanirole(both represented in Figure 10, panels B and C, respectively) were tested by Huang et al., whoreportedthat the final effect of such activity on ALS models was an increase in motor neuron survival [53]. Another agonist for the “D2-like family” of dopamine receptors is the R(+) enantiomer of the Parkinson’s disease drug Pramipexole, known as Dexpramipexole (Figure 10, panel D).

**Figure 11:**
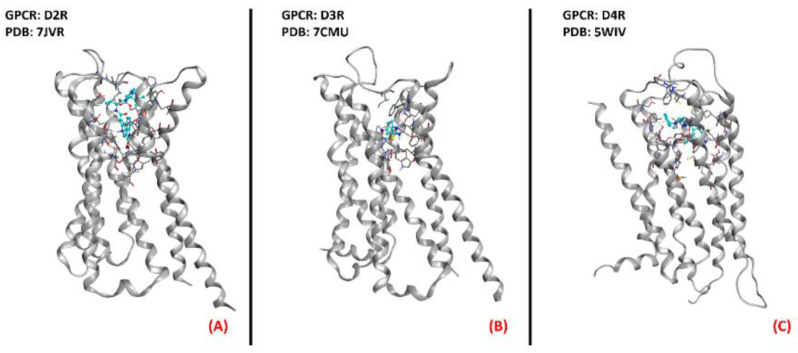
Representation of the three-dimensional structures of the dopamine receptors thatcould be considered for ALS treatment. (**A**) D2R (sourced the Protein Data Bank, PDB code: 7JVR [115], method: cryo-EM, resolution: 2.80 Å), (**B**) D3R (sourced from the Protein Data Bank, PDB code: 7CMU [116], method: cryo-EM, resolution: 3.00 Å), and (**C**) D4R (sourced from the Protein Data Bank, PDB code: 5WIV [117], method: X-ray diffraction, resolution: 2.14 Å). All of the images were created and rendered with MOE.

Even if Pramipexole is a powerful agonist of D2R, D3R, and D4R (all depicted in Figure 11) [55], its R(+) enantiomer has a very low affinity for dopamine receptors, so its neuroprotective effects have to bedue to a non-dopaminergic action [56]. This molecule has been considered a promising candidate for ALS conditions [57]. After the phase III clinical trial, however, its development in Europe was discontinued [58].

D2R is still a very relevant target for Parkinson’s disease treatment, but the presented literature concords in considering it also a protein of high therapeutic potential for treating ALS.

### 2.5. Serotonin (5-HT) Receptors

**Figure 12:**
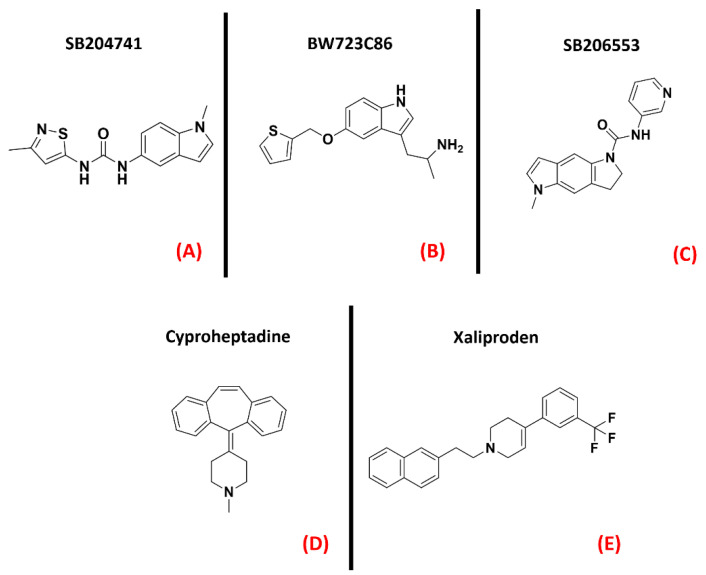
The chemical structures of the serotonin receptor modulators are treated in this review. The selective 5-HT_2B_ antagonist SB204741 (**A**), the 5-HT_2B_ agonist BW723C86 (**B**). (**C**) Structure of the 5-HT_2B/C_ mixed inverse agonistSB206553; (**D**) chemical structure of Cyproheptadine, a non-selective mixed serotonin receptor antagonist (which is an inverse agonist of 5-HT_2B_). (**E**) Structureof the 5-HT_1A_ receptor agonist Xaliproden.

Serotonin (also called 5-hydroxytryptamine, or 5-HT) receptors represent one of the most populated subfamilies of class AGPCRs, consisting of13 G-protein coupled receptor isoforms (5-HT_1A_, 5-HT_1B_, 5-HT_1D_, 5-HT_1E_, 5-HT_1F_, 5-HT_2A_, 5-HT_2B_, 5-HT_2C_, 5-HT_4_, 5-HT_5A_, 5-HT_5B_, 5-HT_6_, and 5-HT_7_) distributed throughout the entire human organism, and a cation channel (5-HT_3_), mainly involved in gastrointestinal motility [118].It is also interesting to note that almost all of these isoforms are present in the CNS [119]. Concerning ALS, the serotonin receptor which has gained the greatest popularity is 5-HT_2B_. An article from Oussini et al., reportedthatthe activity of this biological entity could limit the degeneration of spinal cord mononuclear phagocytes, which is a process typical of neurodegenerative diseases. This article highlighted that the ablation of the 5-HT_2B_ gene resulted in an acceleration of ALS progression in mutant SOD1 mouse models. Indeed, they showed that the administration of a 5-HT_2B_ selective antagonist (SB204741, Figure 12, panel A) caused an important reduction in microglia viability, while treatment with the agonist BW723C86 (Figure 12, panel B) induced an increase in viability [59]. Another work by Dentel et al., reported that the spasticity associated with ALS progression couldbe strongly alleviated by the administration of inverse agonists of 5-HT_2B/C_ such as SB206553and Cyproheptadine (both depicted in Figure 12, panels C and D, respectively) [60]. A recent article by Arnoux et al., on the other hand, highlighted the lack of beneficial effects when ALS-affected SOD1^G86R^ mutants weretreated with the 5-HT_2B_ agonist BW723C86 [61]. The main factor that has always limited the development of 5-HT_2B_ agonists is their inherent cardiotoxicity, which can determine valvular heart disease [62]. Two randomized, double-blind, placebo-controlled multicenter studies (phase III) were conducted in 2004 by Meininger et al. to evaluate the potential benefits of Xaliproden (Figure 12, panel E), a 5-HT_1A_ receptor agonist with neuroprotective effects, in ALS patients.

**Figure 13:**
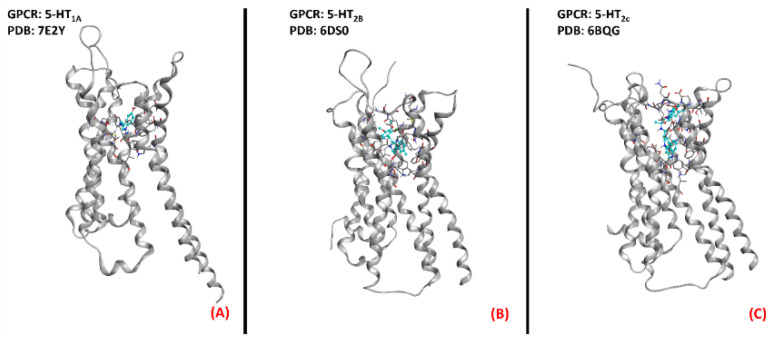
Three-dimensional structures of the serotonin receptors that could be considered for ALS treatment. (**A**) 5-HT_1A_ (sourced from the Protein Data Bank, PDB code:7E2Y [120], method: cryo-EM, resolution: 3.00 Å), (**B**) 5-HT_2B_ (sourced from the Protein Data Bank, PDB code: 6DS0 [121], method: X-ray diffraction, resolution: 3.19 Å), and (**C**) 5-HT_2C_ (sourced from the Protein Data Bank, PDB code: 6BQG [122], method: X-ray diffraction, resolution: 3.00 Å). All of the images were created and rendered with MOE.

Despite the promising outcomes of the prior experiments [63], no effective slowing down in the progression of the pathology was evidenced. Even if some limitations have been encountered in serotoninergic modulation for motor neuron disease, the 5-HT receptorshave been demonstrated to be targets of relevance in the ALS scenario. The three-dimensional structures of 5-HT_1A_, 5-HT_2B_, and 5-HT_2C_ are represented in Figure 13.

### 2.6. GPR17 Receptor

**Figure 14:**
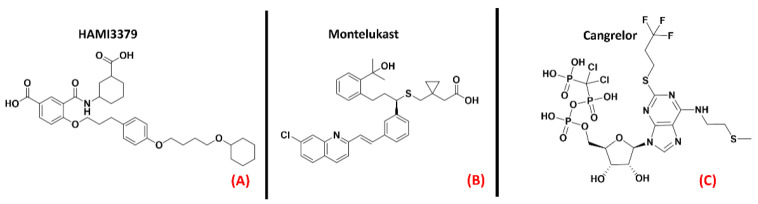
The chemical structures of the GPR17 inhibitors. The non-selective inhibitors HAMI3397, Montelukast (sold as a CysLT receptors inhibitor for asthma), and Cangrelor (an antiplatelet drug, reversible inhibitor of P2Y_12_ receptor), respectively (**A**–**C**).

GPR17 (also known as “uracil nucleotide/cysteinyl leukotriene receptor”) is a protein belongingto the 15th subfamily of classA GPCRs. One of its peculiarities is that its structure is phylogenetically related to both cysteinyl leukotriene (CysLT) receptors and to purinergic P2Y receptors [123]. This receptor is activated by uracil nucleotides such as UDP, UDP-glucose, and UDP-galactose, but is also sensitive to CysLTs, like Leukotriene D4 and C4 [124]. GPR17 is mainly expressed in the CNS (but also in kidneys, heart, and generally in organs that can experience ischemic damage), and a more pronounced presence of this protein has been highlighted in oligodendrocyte precursor cells (OPCs). Upregulation of GPR17 can be observed in neuronal cells surrounding an ischemic-injured area, making this protein a marker for cellular stress and death.It has been reported in the literature that in the case of a demyelinating event, GPR17 is involved in the remyelination process, but the mechanismof its involvement is still debated [125].What is known is thatGPR17 is deputed to accompany the OPCs in the early stages of their differentiation process, and so its downregulation is necessary for these cells to complete their maturation. As a result of this, overexpression of this protein leads to incomplete OPC development, impairing myelination and promoting inflammatory responses [64].Moreover, GPR17 upregulation inneurons was linked to increased cell damage by Zhao et al., who also observed that its knockdown attenuated neuronal injury and microgliosis [65]. In the field of ALS, GPR17 wasdemonstrated by Bonfanti et al., to be upregulated in the spinal cord of SOD1^G93A^ mouse models [66]. As reported in a recent study by Raffaele et al., whilethe application of non-selective GPR17 antagonists such asHAMI3379 (Figure 14, panel A) or Montelukast (a marketed CysLT receptor inhibitor, which is represented in Figure 14, panel B) has been shown to improve remyelination processes (as also demonstrated by Merten et al. for the first of these two molecules [67]), the implementation of agonists has also been demonstrated to be beneficial in pushing OPCs to start differentiating [68]. Jin et al., asserted that the inhibition of GPR17 by Cangrelor (Figure 14, panel C) results in the amelioration of cognitive deficits through the inhibition of oxidative stress and neuroinflammation in Alzheimer’s Disease mouse models [69].

Marschallinger et al., in a recent paper, highlighted a restoration in cognitive function and a reduction in neuroinflammation in rats treated with Montelukast [70].Another study by Burnstock et al., indicated that the in vivo knockdown of GPR17 markedly reduced brain damage [71].

**Figure 15:**
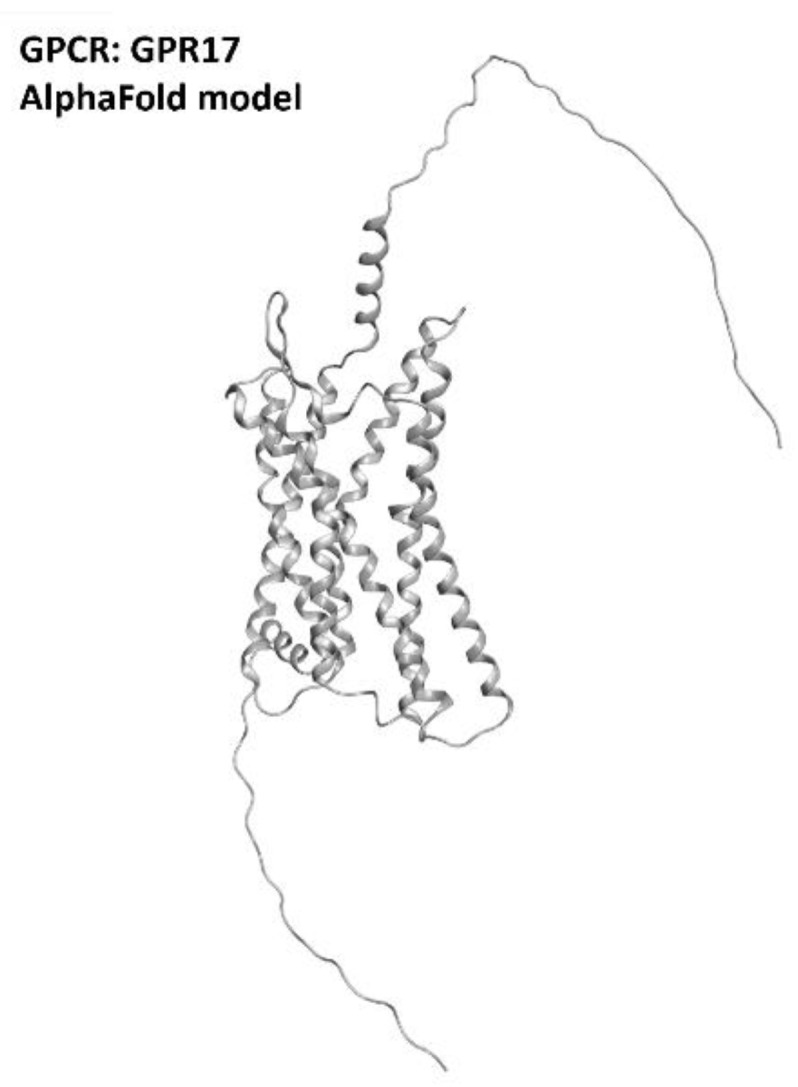
Structure of the GPR17 receptors (with no experimentally resolved structure available, the model sourced from the AlphaFold database is presented). The image was created and rendered with MOE.

The information available nowadays convergesin indicating GPR17 (which three-dimensiona structure is provided in Figure 15) as a promising target for neuroinflammation and neurodegeneration diseases. Even if, at the present moment, theseefforts are more focused on multiple sclerosis treatment, GPR17 regulation for ALS is also attracting increasing interest from the scientific community.

### 2.7. Adrenergic Receptor β_2_

**Figure 16:**
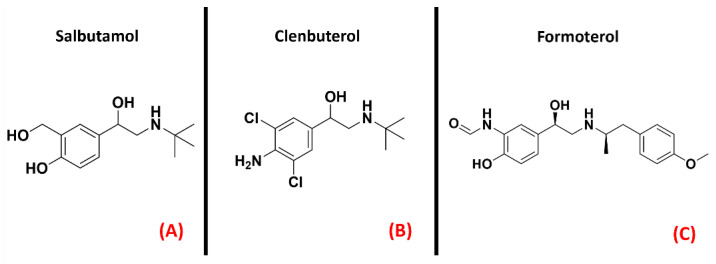
The chemical structures of the adrenergic β_2_ receptor agonists Salbutamol (**A**), Clenbuterol (**B**), and Formoterol (**C**).

Adrenergic receptors are part of the 17th subfamily of classA GPCRs and are divided into ninedifferent isoforms (α_1A_, α_1B_, α_1D_, α_2A_, α_2B_, α_2C_, β_1_, β_2_, β_3_), which are involved in very important physiological functions such as smooth muscle contraction and relaxation, heart muscle contraction (mainly receptors β_1_ and β_2_) [126], and glycogenolysis [127]. The research to find a link between adrenergic transmission and ALS has been focused on the β_2_ isoforms of this GPCR. Historically, β_2_ agonists represent one of the main solutions for asthma therapy [128], and drugs such as Salbutamol, Clenbuterol, and Formoterol (all depicted in Figure 16) are an example of this. A recentwork by Bartus et al. highlighted the potentialities of β_2_ agonists for ALS, reporting that the downstream effects of these molecules can be useful for protecting spinal cord neurons, both preserving and/or restoring their function [72].

**Figure 17:**
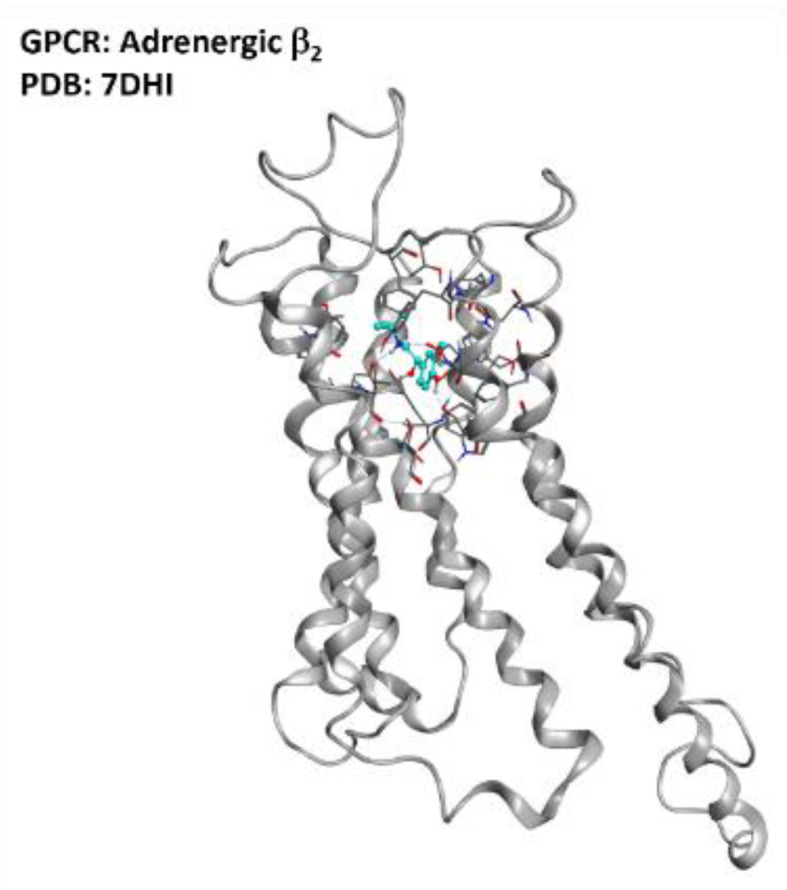
Representation of the structure of the adrenergic β_2_ receptor (sourced from the Protein Data Bank, PDB code: 7DHI [129], method: cryo-EM, resolution: 3.26 Å). The image was created and rendered with MOE.

The pathways responsible for such outcomes presented by Bartus et al. are very biologically important for cell homeostasis, such as the cAMP/PKA/CREB pathway, the PI3K-Akt-mTOR pathway, and the PKA/SIRT1 pathway. Other than neuroprotection, other effects attributed by the authors to this class of ligands are the increase in muscle strength and the amelioration of mitochondrial function. Another study by Teng et al. reported a favorable effect of the β_2_ agonist Clenbuterol on SOD1^G93A^ mice [73]. The outcomes of these studies open new possibilities in drug discovery for ALS, focusing special attention on adrenergic β_2_ receptor modulation. A three-dimensional representation of the adrenergic β_2_ receptor is provided in Figure 17.

### 2.8. Histamine Receptors

**Figure 18:**
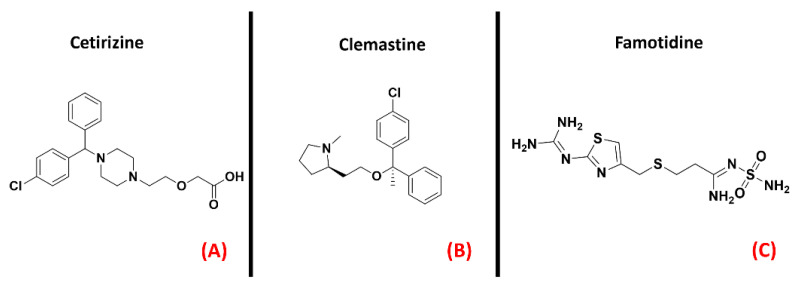
The chemical structures of the H_1_ receptor antagonists Cetirizine (**A**) and Clemastine (**B**). The H_2_ receptor antagonist Famotidine is also reported (**C**).

Histamine receptors represent a group of class AGPCRs thathas attracteda lot of interest inthe pharmaceutical world in recent decades. This family is composed of four different members (H_1_, H_2_, H_3_, and H_4_), each with a specific localization in the organism. Their functions, vary from one isoform to another, ranging from vasoconstriction (H_1_) to gastric acid secretion (H_2_), to neurotransmitter release (H_3_), to immunoregulation (mainly H_2_ and H_4_) [130]. For each histamine receptor, the research has mainly focused on the mechanism of antagonism, of which several marketed drugs are still now relevant examples (e.g., Cetirizine, Figure 18, panel A, for H_1_ antagonism; or Famotidine, represented in Figure 18, panel B, for H_2_ blockage) [131].

**Figure 19:**
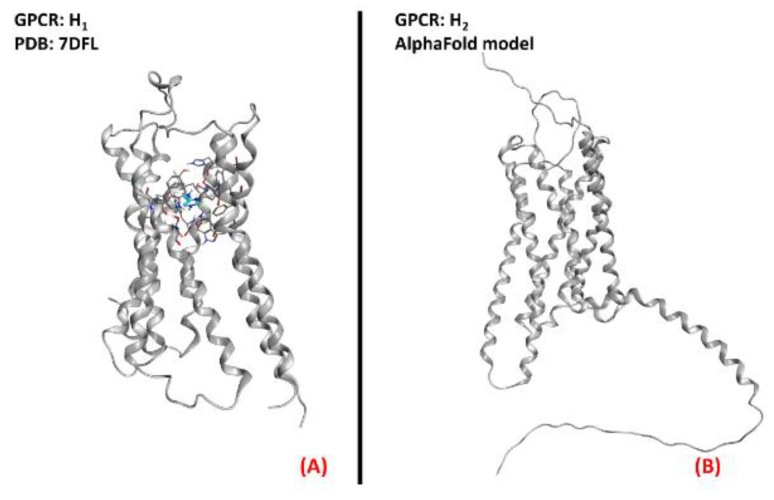
Representation of the three-dimensional structures of the histamine receptors that could be considered for ALS treatment. (**A**) The H_1_ receptor (sourced from the Protein Data Bank, PDB code: 7DFL [132], method: cryo-EM, resolution: 3.30 Å) and (**B**) the H_2_ receptor (with no experimentally resolved structure available, the model sourced from the AlphaFold [133] database is presented).The images were created and rendered with MOE. The H_3_ and H_4_ receptors both lack experimentally resolved structures, but their AlphaFold models are available.

An article from Apolloni et al. reported the involvement of histaminergic signals in ALS progression, highlighting that histamine receptors are dysregulated in the cortex, spinal cord, and hypothalamus of SOD1^G93A^ ALS-affected mice [74]. This study reported that histamine could counteract the pro-inflammatory phenotype of microglia, mainly through its H_1_ and H_4_ receptor isoforms. This would be mediated by both the reduction of NOX-2 and NF-kB expression and the increase in production of other species, such as IL-6 and IL-10. Another work by Volontè et al. highlighted the neuroprotective effects of histamine signaling in ALS, again giving higher relevance to H1R (represented in Figure 19, panel A) and H4R [75]. On the other hand, Zhang et al. described H_1_ and H_4_ receptors as being responsible for pro-inflammatory cytokine release in microglia, while H_2_ and H_3_ were considered to be the main actors of anti-inflammation in that environment (the H_2_ receptor is depicted in its three-dimensional structure in Figure 19, panel B) [76]. Another article by Apolloni et al. reported an amelioration in ALS progression of SOD1^G93A^ mice treated with the anti-histaminergic drug Clemastine [77]. Even if much more remains to be understood about the specific role of each histamine receptor isoform in ALS progression, what is certainis that this GPCR family has already proven to be a promising target for drug development against neuroinflammation and neurodegeneration.

### 2.9. Cannabinoid Receptors

**Figure 20:**
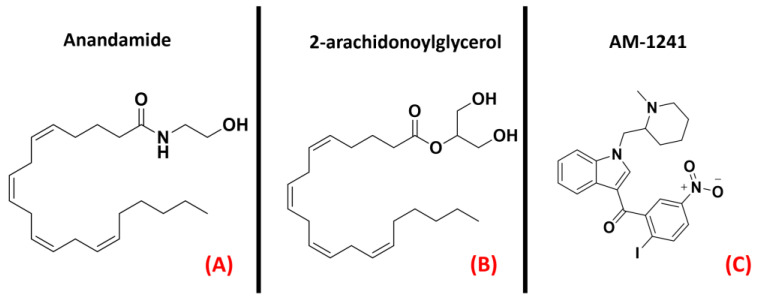
The chemical structures of the endogenous CB_1_ and CB_2_ receptors agonists anandamide (**A**) and 2-arachidonoylglycerol (**B**). The selective CB_2_ receptor agonist AM-1241 is also reported (**C**).

A group of class AGPCRs thatare of high interest at the present date is certainly the cannabinoid receptors. These biological entities are the main actors in the endocannabinoid system, andplayrelevant roles in several physiological processes. Indeed, the first of its two main isoforms, called CB_1_, is mainly located in both the central and peripheral nervous system, acting as a neurotransmitter release modulator in response to the binding of its agonists (mainly anandamide, but also 2-arachidonoylglycerol, both represented in Figure 20, panels A and B, respectively). In the majority of cases, CB_1_ is coupled with G_i/o_ protein, leading to adenylyl cyclase inhibition and consequent decrease in cAMP upon activation. The final effect of such an action is the reduction of neurotransmitter release in the synapse. On the other hand, the CB_2_ receptor is mainly localized on the surface of the immune system cells. Its main agonist is 2-arachidonoylgycerol, binding of which leads to the inhibition of adenylyl cyclase through G_i/o_ subunit action [134]. The final main effect is immunosuppression [135]. As reported by an article from Giacoppo and Mazzon, several studies have shownhow the application of cannabinoid receptor agonists in SOD1^G93A^ mouse models of ALS could be beneficial for the neuroprotective effects mediated by them [78]. Similarly, in 2019,Urbi et al. performeda meta-analysis on the studies regarding the application of cannabinoids in ALS murine models, highlighting the effective concordance in assessing that their application leads to a delay in disease progression [79]. A study by Shoemaker et al. highlighted that the increase in survival could be more addressable to the CB_2_ isoform, showing that the administration of the CB_2_ selective agonist AM-1241 (Figure 20, panel C) increased survival by 56% [80]. This molecule was also examined for cannabinoid-mediated ALS treatment by Kim et al., with similar results [81].

**Figure 21:**
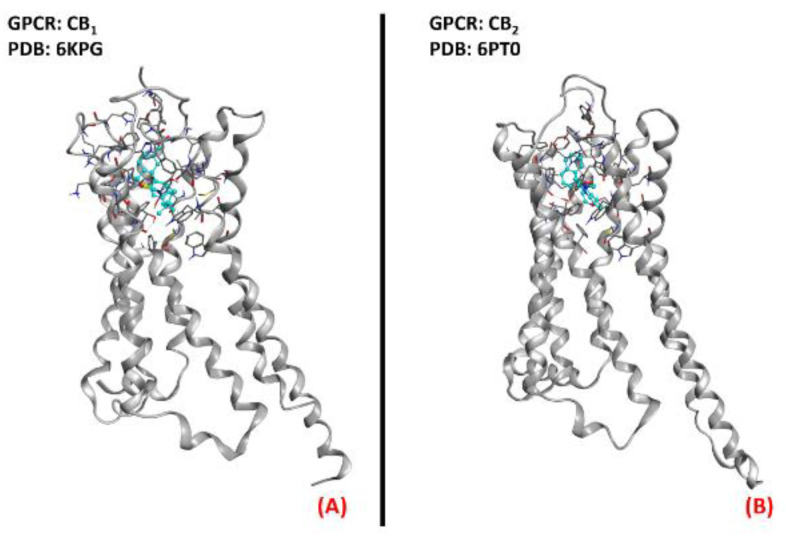
The structures of (**A**) the CB_1_ receptor (sourced from the Protein Data Bank, PDB code: 6KPG [137], method: cryo-EM, resolution: 3.00 Å) and (**B**) the CB_2_ receptor (sourced from the Protein Data Bank, PDB code: 6PT0 [138], method: cryo-EM, resolution: 3.20 Å). The images were created and rendered with MOE.

In addition to this, Bilsland et al. reported that the knock-out of CB_1_ receptors in SOD1^G39A^ ALS-affected mice had no appreciable effect on disease onset [136], and regarding this, Shoemaker et al. reportedthat the activation of CB_1_ could exacerbate disease progression [80]. The literature availabletoday regarding the application of molecules acting on the endocannabinoid system for ALS treatment converges in the possible evaluation of a therapy based on CB_2_ selective agonists.A three-dimensional representation of both CB_1_ and CB_2_ receptorsis provided in Figure 21.

### 2.10. Prostaglandin E_2_ Receptor (PGE2R)

**Figure 22:**
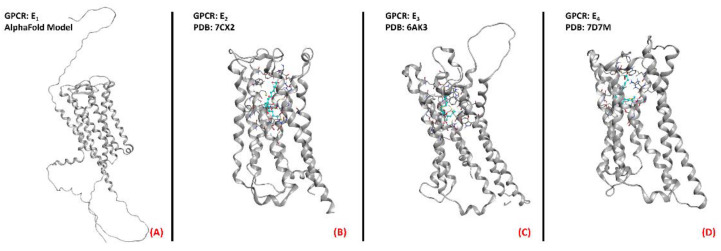
Structure ofthe PGE_2_ receptors (**A**) EP_1_ (with no experimentally resolved structure available, the model sourced from the AlphaFold database is presented), (**B**) EP_2_ (sourced from the Protein Data Bank, PDB code: 7CX2 [149], method: cryo-EM, resolution: 2.80 Å), (**C**) EP_3_ (sourced from the Protein Data Bank, PDB code: 6AK3 [150], method: X-ray diffraction, resolution: 2.90 Å), and (**D**) EP_4_ (sourced from the Protein Data Bank, PDB code: 7D7M [151], method: cryo-EM, resolution: 3.30 Å). All of the images were created and rendered with MOE.

Prostaglandin E_2_ receptors (PGE_2_) are a series of class A GPCRs that selectively bind to prostaglandin E_2_ (also known as dinoprostone), an endogenous arachidonic acid derivative of high importance for several physiological functions. PGE_2_ can bedivided into fourisoforms, named E_1_, E_2_, E_3_, and E_4_ (all represented in their three-dimensional structure in Figure 22). With the exception of the first, which stimulates phospholipase C if agonized, the other isoforms act on adenylyl cyclase and, specifically, the EP_2_ and EP_4_ isoforms (coupled with a G_s_ subunit) stimulate its function whenagonized, while EP_3_ inhibits AC through its action (being coupled to a G_i/o_ subunit) [139]. EP_1_ function has been correlated with hyperalgesia [140], immunoregulation [141], and colon cancer progression [142]. EP_2_, which is active in the reproductive, visual, cardiovascular, skeletal, and nervous systems, has also been strictly related to tumor promotion, as highlighted in a 2018 study bySun and Li [143]. Minor correlations with cancer have been reported for EP_3_, which is also important for a large variety of functions, ranging from digestion [144] to blood pressure [145] and clotting [146], in addition to pain management [147]. The spectrum of systems in which the fourth isoform of PGE_2_ receptors, EP_4_, is involved is also very wide. Additionally, in this case, EP_4_ has been reported to be hyper-expressed in various types of cancer, mainly prostate cancer [148]. Talking about ALS onset and progression, Iłzecka found increased levels of PGE_2_ in the cerebrospinal fluid of ALS patients, and thereforeconcludedthat this mediator could play a role in disease progression, suggesting that its inhibition could be beneficial [82]. Additionally, Kosuge et al., highlighted the role of PGE_2_ in the ROS generation pathway, focusing on its impact on ALS conditions. The same conclusion was reported in 2008 by Liang et al., whosuggested EP_2_ inhibition as a novel way to treat the neuroinflammation typical inALS [83]. On the other hand, PGE_2_ receptors were proposed to have an unexpected neuroprotective effect on motor neurons by Bilak et al., whoreportedthat the neuroinflammatory process typical of ALS wasmainly due to COX-2-mediated, prostaglandin-independent processes [84]. Taken together, all of these studies converge in evaluating PGE_2_ receptors as interesting pharmacological targets for ALS, being strongly correlated with the significantneuroinflammation characterizing the pathology.

### 2.11. Vasoactive Intestinal Peptide Receptors

**Figure 23:**
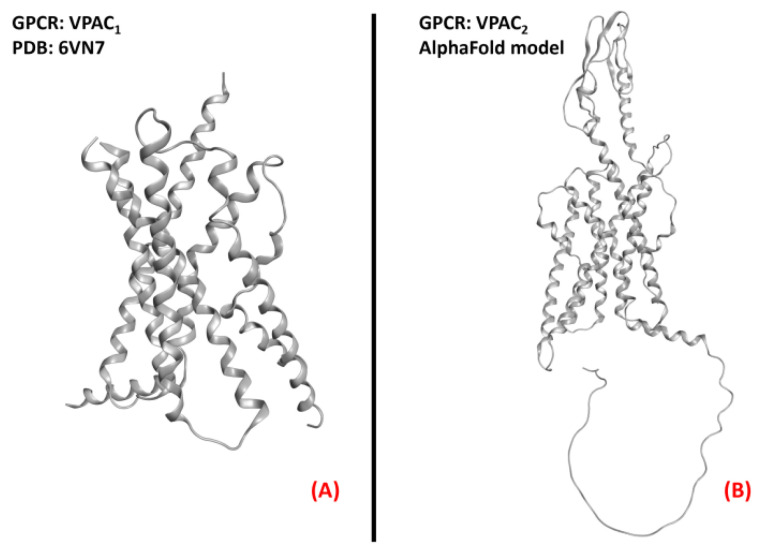
Structure of the receptors (**A**) VPAC_1_ (sourced from the Protein Data Bank, PDB code: 6VN7 [155], method: cryo-EM, resolution: 3.20 Å), and (**B**) VPAC_2_ (with no experimentally resolved structure available, the model sourced from the AlphaFold database is presented). The images were created and rendered with MOE.

The receptors for the vasoactive intestinal peptide are part of the first subfamily of class B GPCRs. As part of this group of proteins, these receptors are responsive to signals mediated by the peptide hormone VIP (vasoactive intestinal polypeptide), formed by 28 amino acids and belonging to the glucagon/secretin superfamily [152]. After being produced by organs such as the gut, pancreas, and brain, VIP act in different physiological functions depending on the target tissue and the receptor isoform interacting with it. Indeed, two vasoactive intestinal polypeptide receptor isoforms are known, namely VPAC_1_ and VPAC_2_ (both represented in Figure 23). Both of these proteins are highlyexpressed throughout the human body, from the smooth muscle of the GI tract and blood vessels to the reproductive system, lungs, spleen, and brain [153]. Their activity is mediated by a G_s_ protein, and involves the activation of adenylyl cyclase upon binding to VIP, consequently activating the protein kinase A (PKA) [154].

The implication of VIP in the CNS has been noticed whenstudying both the circadian rhythm and schizophrenia [153], but its importance as a potential target for ALS therapy is of more recent discovery. In 2008, Staines considered the possibility of studying vasoactive neuropeptides for degenerating pathologies such as MS and ALS [85], and a previous article by Iwasaki et al. specifically referred to the neurotrophic properties exerted by VIP in the degenerating diseases of motor neurons [86]. Solés-Tarrés et al. analyzed the neuroprotective effects of both VIP and PACAP (pituitary adenylate cyclase-activating polypeptide, a peptide hormone binding to both VPACs and PACAP receptors), also evaluating the synthetic derivatives available to mimic their action, with a special focus on the intrinsic pharmacokinetic problems of these species [87]. Waschek already identifiedboth VIP and PACAP as promising targets for neuroinflammation in the CNS [88], as pointed out again in recent work by Martinez et al. [89].

The interaction with receptors of vasoactive peptides has been demonstrated to be a promising way to counteract the neuroinflammatory and degenerative effects of ALS, mainly through biological and/or chemical species mimicking the functions of the endogenous peptides. Further research on thistopic will define the best way to accomplish this task.

### 2.12. Metabotropic Glutamate Receptors (mGluRs)

Metabotropic glutamate receptors (mGluRs) are GPCRs belongingto class C of this family of proteins. As the name suggests, these entities bind to the neurotransmitter glutamate, exerting different functions in both the central and peripheral nervous systems. They can be divided into threegroups, with the first beingcomposed of mGluR1 and mGluR5, predominantly postsynaptic, which, once activated, cause the stimulation of phospholipase C (PLC) through G_q_-mediated signaling. Group II (formed by mGluRs 2 and 3) and III (of which mGluRs 4, 6, 7, and 8 are a part) receptors are mainly presynaptic and are all coupled with a G_i/0_ subunit, which inhibits the activation of adenylyl cyclase, causing presynaptic inhibition. The functions of this family of proteins are majorly related to the nervous system, from the modulation of neurotransmission (e.g., gabaergic and dopaminergic) and of other proteins’ signaling (e.g., NMDA receptors), to synaptic plasticity regulation [156]. This being said, it appears clear that the possibility of their involvement in ALS onset and progression is more than possible. Anneser et al. found a strong upregulation of mGluRs in the spinal cord with ALS, leading to the propagation of glial proliferation [90]. Hyperactivity of group I mGluRs has been correlated with neuroinflammation. Indeed, as demonstrated by Milanese et al., SOD1^G93A^ ALS-affected mice with mGluR1 knockdown experience a reduction in microglia and astrocyte activation, decreasingmitochondrial damage and improving survival [91], and this phenomenon was also highlighted by Rossi et al. [92].

**Figure 24:**
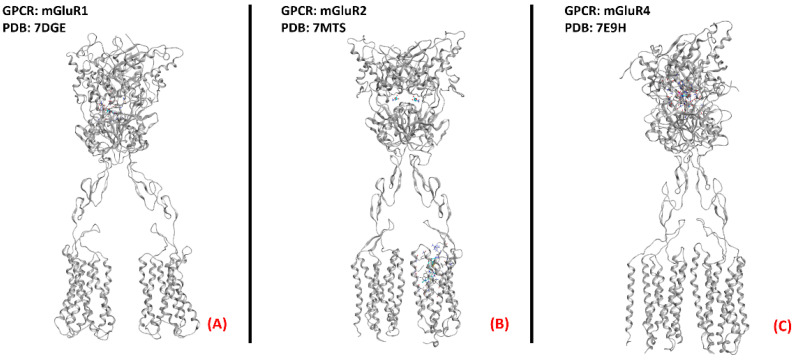
One example of each group of the metabotropic glutamate receptors. (**A**) mGluR1, a member of the first group of mGluRs (sourced from the Protein Data Bank, PDB code: 7DGE [157], method: cryo-EM, resolution: 3.65 Å), (**B**) mGluR2 (owing to mGluRs group II, sourced from the Protein Data Bank, PDB code: 7MTS [158], method: cryo-EM, resolution: 3.20 Å), and (**C**) mGluR4, part of group III of the mGluRs (sourced from the Protein Data Bank, PDB code: 7E9H [159], method: cryo-EM, resolution: 4.00 Å). All of the images were created and rendered with MOE.

Anneser et al. showed the beneficial and protective effects of both agonism and antagonism of group I mGluRs for motor neuron disease, while less promising effects were derived from modulation of other mGluRs [93]. Crupi et al. recently pointed out that the beneficial therapeutic modulation of mGluRs is usually achieved throughthe reduction of the excitotoxicity drive via mGluR I inhibition or mGluR II and III agonism [94]. In conclusion, the literature supports the possibility of investing resources in the treatment of motor neuron diseases via mGluR modulation.A three-dimensional representation of mGluR1, mGluR2, and mGluR4 receptorsis provided in Figure 24.

## 3. Conclusions

In this review, we provideda panoramic view of the involvement of different G-protein-coupled receptors in the onset and progression of ALS, evaluating what has already been discovered on these biological entities, and highlighting what the next steps in researchcould be, alwayson the basis of the present literature on the topic. Our analysis shows that a GPCR-based therapy for ALS could be considered a practical possibility for the eradication of the ALS condition, and we encourage scientific groups all around the world in directingefforts towardsthis field.

## Figures and Tables

**Table 1:** Table reporting the different smallmolecules currentlyin FDA clinical trials for ALS treatment (updated 13 April 2022).

Molecule	Target/Mechanism	Developer	Clinical Phase
Ibudilast	Macrophage migration inhibitory factor inhibitor	MediciNova	Phase II/III
Prosetin	Mitogen-activated protein kinase inhibitor	ProJenX	Phase I
Sotuletinib	Macrophage colony-stimulating factor receptor antagonist	Novartis	Phase II
EPI 589	NAD(P)H dehydrogenase modulator	PTC Therapeutics	Phase II
DNL 343	Eukaryoticinitiationfactor2b stimulant	Denali Therapeutics Inc	Phase I
Celecoxib/ciprofloxacin	Cyclo-oxygenase 2 inhibitors/DNA gyrase inhibitors	NeuroSense Therapeutics	Phase I
Fingolimod	Apoptosis stimulant and immunosuppressant	ALS Therapy Development Institute	Phase II
Trehalose	Autophagy stimulant and protein aggregation inhibitor	Massachusetts General Hospital	Phase II/III
Sodium cromoglicate	Glial cell modulator and mast cell stabilizer	AZTherapies	Phase II
Dexpramipexole	Antioxidant and apoptosis inhibitor	Knopp Biosciences	Phase II
Masitinib	Tyrosine kinase inhibitor	AB Science	Phase III
NP 001	Macrophage modulator	Neuvivo	Phase II
Fasudil	Rho-associated kinase inhibitor and vasodilatator	Woolsey Pharmaceuticals	Phase II
Levosimendan	Calcium-sensitising phosphodiesterase inhibitor and potassium channel agonist	Orion	Phase III
Apilimoddimesylate	Interleukin 12 inhibitor and interleukin 23 inhibitor	AI Therapeutics	Phase II
Verdiperstat	Peroxidase inhibitor	Biohaven Pharmaceuticals	Phase II/III
Pridopidine	Sigma-1 receptor agonist	Massachusetts General Hospital, Prilenia Therapeutics	Phase II/III
Triheptanoin	Triglyceride replacement agent	Ultragenyx Pharmaceutical	Phase I/II
Reldesemtiv	Troponin stimulant	Cytokinetics	Phase III
BIIB 100	Exportin-1 protein inhibitor	Biogen	Phase I
AGX 201	Histamine receptor modulator	AgoneX Biopharmaceuticals	Phase I
Ranolazine extended release	Sodium channel antagonist	Gilead Sciences	Phase II
GDC 0134	Mitogen-activated protein kinase 12 inhibitor	Genentech	Phase I
NPT520 34	Phosphatidylinositol 3 kinase modulator	Neuropore Therapies	Phase I

**Table 2:** Table summarizing the evidence about the therapeutic potential of the GPCRs examined in this article for ALS treatment.

Receptor/Receptor Family	Cellular Expression	Potential for ALS Treatment	References
Adenosine receptors	Circulatory, immune, respiratory, and nervous systems	Ambiguous	[30,31,32,33,34,35,36,37]
Purinergic receptors P2Y	Almost all human tissues	Antagonism	[38,39,40,41,42,43]
Chemokine receptors	Predominantly on leukocytes surface	CXCR3, CXCR4, and CCR2 Antagonism	[44,45,46,47]
Angiotensin II receptors	Adrenal cortex, kidneys, vascular and cardiac muscles, nervous system	AT_1_ Antagonism	[48,49,50]
Dopamine receptors	Arteries, heart, kidneys, CNS	D2R Agonism	[51,52,53,54,55,56,57,58]
Serotonin receptors	Almost all human tissues	Ambiguous	[59,60,61,62,63]
GPR17 receptor	CNS, kidneys, heart	Antagonism	[64,65,66,67,68,69,70,71]
Adrenergic receptor β_2_	GI tract, respiratory system, blood vessels, pancreas, nervous system	Agonism	[72,73]
Histamine receptors	GI tract, circulatory, immune, and nervous systems.	Ambiguous	[74,75,76,77]
Cannabinoid receptors	CNS and immune system	CB_2_ agonism	[78,79,80,81]
Prostaglandin E_2_ receptor	GI tract, kidneys, reproductive, skeletal, immune, and nervous systems.	Ambiguous	[82,83,84]
Vasoactive intestinal peptide receptors	Almost all human tissues	Agonism	[85,86,87,88,89]
Metabotropic glutamate receptors	Nervous system	mGluR I antagonism/mGluR II and mGluR III agonism	[90,91,92,93,94]
